# Supplementary figures and images for: Functional analysis of ARF1 from Cymbidium goeringii in IAA response during leaf development
Source: PeerJ. 2022 Mar 10;10:e13077. doi: 10.7717/peerj.13077 (PMC8918147; doi:10.7717/peerj.13077)

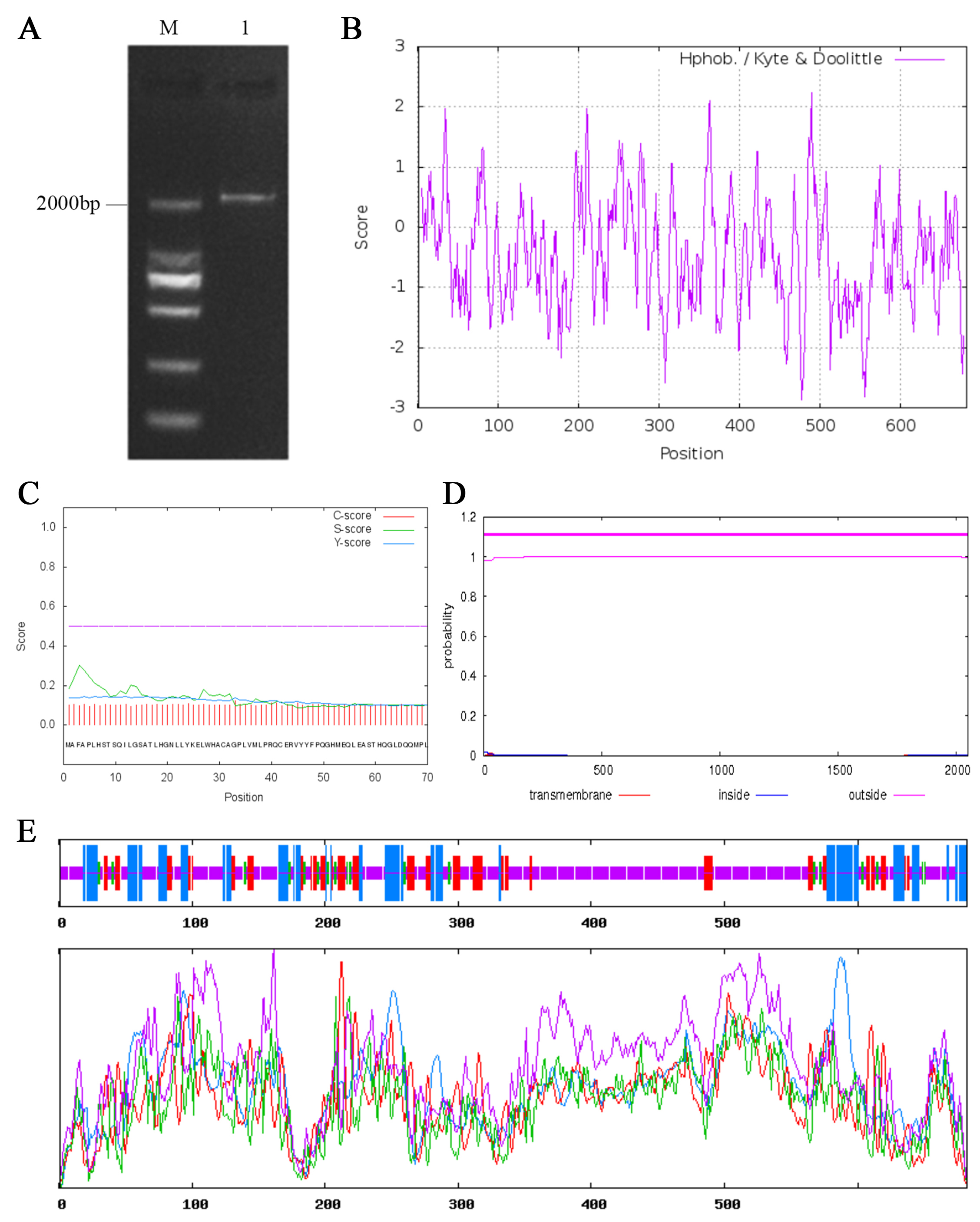

Supplement: Supplemental Information 3 — (A) Electrophoresis image of PCR amplification. M: DL2000 Marker; 1: CgARF1. (B) Hydrophobic properties prediction by ExPASy. (C) Signal peptides prediction by SignalP 4.0. (D) Transmembrane domains prediction by TMHMM2.0. (E) Secondary structure prediction by SOPMA. [file peerj-10-13077-s003.png]

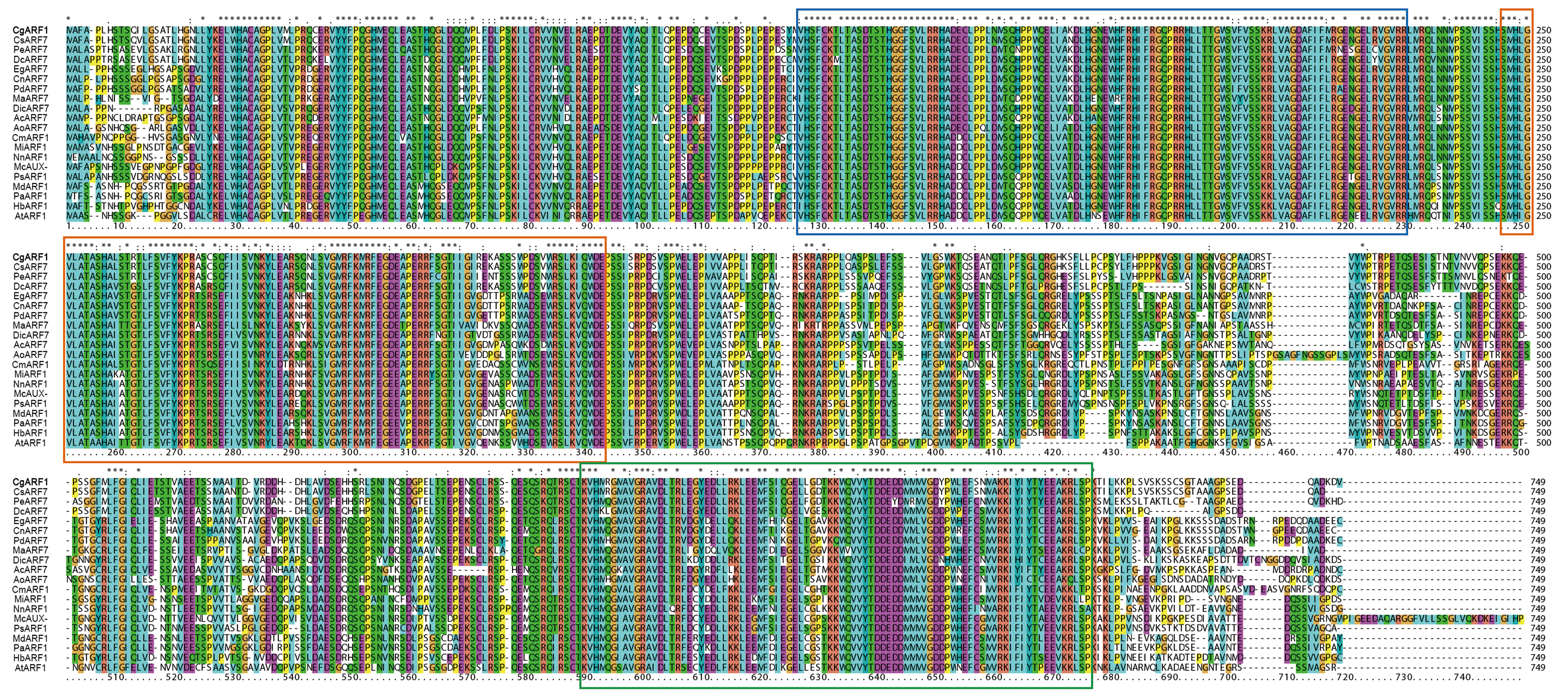

Supplement: Supplemental Information 4 [file peerj-10-13077-s004.png]

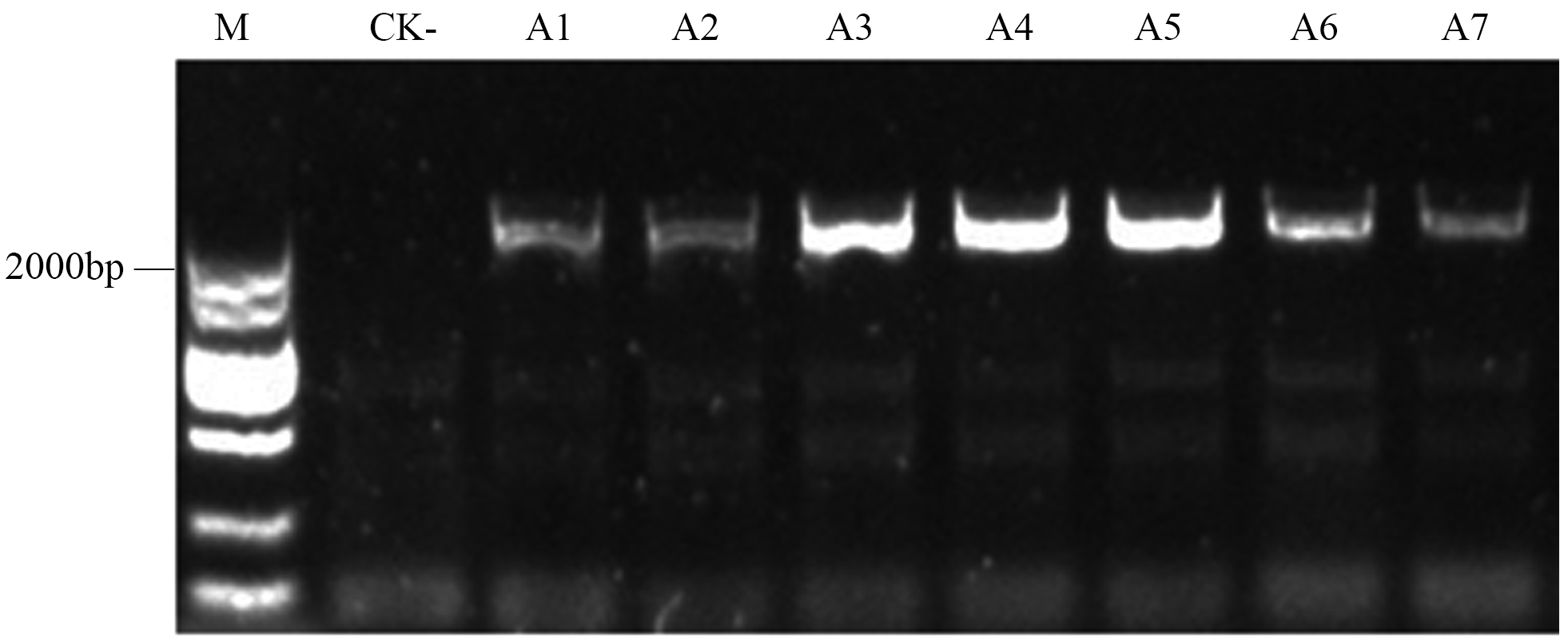

Supplement: Supplemental Information 5 — M: DL2000 Marker; CK-: the test sample of WT. A1-A9: test samples of transgenic lines. [file peerj-10-13077-s005.png]
